# Metastatic gastric tube cancer detected in a resected mandibular bone with osteoradionecrosis

**DOI:** 10.1259/bjrcr.20150192

**Published:** 2015-08-11

**Authors:** Joe Iwanaga, Osamu Iwamoto, Keita Todoroki, Ryuchiro Tanoue, Akihiro Koba, Jingo Kusukawa

**Affiliations:** Dental and Oral Medical Center, Kurume University School of Medicine, Kurume, Japan

## Abstract

Osteoradionecrosis (ORN) of the jaw is an intractable complication of radiotherapy for head and neck cancer. However, osteolytic lesions are often hard to distinguish from malignancies. We report a rare case of metastatic cancer in a resected mandibular bone after segmental resection for ORN of the jaw. A 63-year-old male with a history of subtotal oesophageal resection for oesophageal cancer and reconstruction of the oesophagus with a gastric tube subsequently developed ORN of the jaw. Conservative treatment was unsuccessful and pathological fracture of the necrotic mandible occurred. The patient underwent segmental resection of the mandible and adenocarcinoma was detected in the resected mandibular bone. Immunohistochemical staining for cytokeratin 7 and 20 revealed that the adenocarcinoma had metastasized from the reconstructed gastric tube. This case highlights the fact that cancers of the gastric tube may metastasize to radiation-induced necrotic bone tissue in the mandible.

Osteoradionecrosis (ORN) of the jaw is an intractable complication of radiotherapy for head and neck cancers, affecting around 2% of all patients exposed to radiation.^1^ ORN is an intractable and progressive disease, conservative therapy is generally the first-choice treatment, and segmental resection of the mandible may be considered as second-line therapy rather than conservative treatment.

Although metastatic tumours account for only 1–3% of all malignancies in the oral and the maxillofacial region, the mandible is the main metastatic site.^2,3^ However, there have been no previous reports of metastasis to a jaw bone affected by ORN.

We report a rare case of metastasis from a reconstructed gastric tube detected in the resected bone after segmental resection for ORN of the jaw. This study was performed in keeping with the requirements of the Declaration of Helsinki (64th World Medical Association General Assembly, Fortaleza, Brazil, October 2013).

## Clinical presentation

A 63-year-old male was referred to our department with a chief complaint of trismus in September 2009. His past history included subtotal oesophageal resection for oesophageal cancer and reconstruction of the oesophagus with a gastric tube in 1999. Radical neck dissection and postoperative radiotherapy (total 74.8 Gy) were carried out in 2003 for cervical lymph node metastasis, and *de novo* cancer in the reconstructed gastric tube was treated by argon plasma coagulation in 2010. He had no medical history of bisphosphonates formulation, such as internal medicine or intravenous injection.

## Treatment

**Figure 1.**
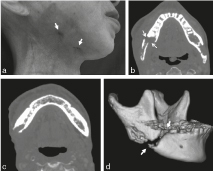
Preoperative images of the mandible. (a) Two fistulae (arrows) draining pus were recognized at the marginal mandibular line. (b) A pathological fracture (arrows) of the right side of the mandible was recognized (c) but there was no evidence of tumours around the mandibular canal. (d) Three-dimensional-CT image of the mandible. A definitive fracture line (arrows) was recognized around the corner of the mandible.

**Figure 2.**
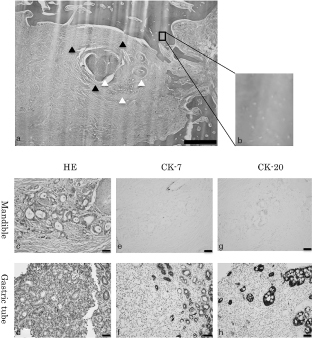
Histopathological images of the resected specimen. (a) HE staining. Adenocarcinoma (white arrowheads) near the inferior alveolar nerve bundle (black arrowheads). Scale bar = 1 mm. (b) Higher magnification of (a) showing acellular osteocytic lacunae in the trabeculae. (c) HE staining of cancer in the mandible. (d) HE staining of cancer in the gastric tube. (e) CK-7 immunostaining of cancer in the mandible. (f) CK-7 immunostaining of cancer in the gastric tube. (g) CK-20 immunostaining of cancer in the mandible. (h) CK-20 immunostaining of cancer in the gastric tube. CK-7, cytokeratin 7; CK-20, cytokeratin 20; HE, haematoxylin and eosin.

**Figure 3.**
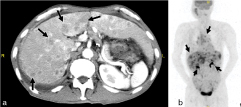
Detailed examination with MDCT and PET. (a) MDCT showed multiple liver metastatic carcinomas (arrows). (b) PET revealed further metastatic carcinomas (arrows) in the lung, liver and adrenal glands. MDCT, multidetector computed tomography; PET, positron emission tomography.

He became aware of a swelling in his left lower gingiva in January 2008, approximately 4 years and 4 months after radiotherapy. He developed severe trismus in August and was referred to our hospital. He was diagnosed with ORN of the jaw and treated with local irrigation, antibiotics, hyperbaric oxygen therapy and sequestrectomy. However, he developed a pathological fracture of the right angle of the necrotic mandible in July 2011 (Figure 1) and underwent segmental resection. Postoperative pathological examination incidentally detected adenocarcinoma in the resected specimen, located at the distal part of the lower right first molar, near the fracture line. Histological examination with haematoxylin -and eosin staining showed a mixture of viable and non-viable bone cells. The bone marrow had been replaced with fibrotic tissue and several cancer cells were present in the mandibular canal (Figure 2). Immunohistochemical staining for cytokeratin 7 and 20 demonstrated that the adenocarcinoma had metastasized from the reconstructed gastric tube (Figure 2). Detailed examination with multidetector CT and positron emission tomography revealed that additional carcinomas metastasized to the lung, liver and adrenal glands (Figure 3). The patient died of multiple organ failure in September 2011.

## Discussion

The reported incidence of ORN after radiation to the head and neck region is approximately 2–3%.^1,4^ Nabil et al^1^ suggested that 70–90% of ORN developed within the first 3 years after radiation, although 4 years and 4 months had elapsed in the current case. It is likely that the patient’s poor oral hygiene as a result of trismus and the high radiation dose contributed to the pathogenesis of ORN.^1^ Owing to the intractable and progressive nature of ORN, it is usually treated surgically, especially in the case of large defects with pathological fractures, which are treated by segmental jaw resection.^5^

Metastatic tumours only account for 1–3% of all malignancies in the oral and maxillofacial region, with breast, lung and kidney cancers being the most frequent primary sites of metastasis to the jaw bone.^2,3^ The ramus and the molar region are the favoured metastatic sites in the mandibular bone. Zhang et al^3^ suggested that good vascularization of the jaw bone increased the risk of metastasis.

The functional relationship between inflammation and development of cancer has been under discussion for a long time.^6^ Although inflammation was not the immediate trigger for the development of cancer in the present case, angiogenesis resulting from chronic inflammation of the mandibular bone could promote metastasis.

The location of the metastatic adenocarcinoma in the present case indicated that the cancer cells likely reached the jaw bone via the inferior alveolar artery. It was difficult to distinguish the necrotic bone from malignancy in the present case because the metastatic tumour was small and located in almost the centre of the resected specimen. Chiandussi et al^7^ also pointed out that osteonecrosis presenting as an osteolytic lesion may be difficult to distinguish from malignancy, while Bedogni et al^8^ reported on jaw metastasis hidden by bisphosphonate-associated osteonecrosis, and concluded that biopsy should be the gold standard for the diagnosis of bone metastases. However, we suggest that, although biopsy would detect larger metastases, it would be less useful in the case of micrometastases.

Of late, the incidence of gastric tube cancer has been increasing because of improvements in the outcome of patients with oesophageal carcinoma. Oki et al^9^ found that the prognosis of resected patients was better than that of non-resected patients. However, no previous studies have reported details of the metastasis of gastric tube cancer to other organs.

## Conclusions

This is the first report of a metastasis to the bone tissue following segmental resection for ORN of the jaw, and also the first report of metastasis from a reconstructed gastric tube to the mandible.

## Learning points

1. The present case highlights the fact that metastatic cancers, including gastric tube cancer, may be present in the resected bone after segmental resection of the mandible, even in patients diagnosed with ORN.
2. Patients with ORN and a history of cancer should be examined carefully to exclude the presence of metastasis.
